# Impact of job demands on police stress response—the roles of basic psychological needs and job autonomy

**DOI:** 10.1186/s12889-022-14758-6

**Published:** 2022-12-05

**Authors:** Pei-feng Chen, Lin Wu

**Affiliations:** School of Public Policy and Administration, Chongqing University, Chongqing, China

**Keywords:** Job demands, Stress response, Basic psychological needs, Job autonomy, Police officers

## Abstract

**Background:**

Police officers are a high-stress group with special job characteristics, and the Chinese police management system places particularly high demands on police officers. Whether the influence of job demands on officers’ job burnout can be deduced to general stress response needs to be verified. Based on the JD-R model, the study aims to explore the impact of job demands on police stress response, whether job autonomy as a job resource has a moderating effect, and whether basic psychological needs mediate this effect.

**Methods:**

A total of 251 police officers in a district-level public security bureau of China, were surveyed using Chinese-language versions of the Job Demands Scale, the Stress Response Scale, the Job Autonomy Scale, and the Basic Psychological Needs Scale. The mediating effect of basic psychological needs and the moderating effect of job autonomy were tested by regression analysis and bootstrap test.

**Results:**

Job demands increase police officers’ stress response, and job autonomy does not play a buffer role but enhances this impact, and job demands can partially reduce the police stress response through the satisfaction of basic psychological needs, that is, there is a masking effect of basic psychological needs.

**Conclusions:**

Adjusting and optimizing the ratio of job demands and autonomy in police work to provide high guidance under high demands is of great value to reduce the negative stress responses among police officers.

## Introduction

As law enforcement officers are regularly exposed to unique stressors during the course of their occupation, they are at elevated risk of developing mental health problems [1, 2], their level of stress response is higher than that of the general population [3]. Police officers are engaged in a stress-intensive occupation full of risks and adverse events. The psychological stress they bear ranks first among all stress occupations [4], so they are known as a “stress group.” With the deepening of social transformation and the emergence of the COVID-19, China’s social security situation is facing a huge test. The law enforcement security risk of the police is increasing. It also puts forward higher demands for the police force in the new period, and the police officers are under increasing stress [5]. Excessive stress not only threatens the physical and mental health of police officers, but also affects professionalism, organizational effectiveness, and public safety [6].

Stress research is focused on the sources and results of stress, namely stressors and stress response [7]. The job characteristics stress model is often used to research the relationship between job characteristics and occupational stress [8]. It is considered that the job characteristics of various occupations are important factors affecting positive (challenge, motivation) and negative (anxiety, worry, depression, impulse) responses [9]. The job demands-resource model (JD-R) is the most widely used job characteristics stress model and the mainstream theoretical model of occupational health psychology. This theory was initially used to explain job burnout, and the job characteristics affecting burnout were divided into job demands and resources [10]. Later, it was widely used in the research of happiness, job engagement [11], and other fields. However, burnout is a prolonged response to chronic emotional and interpersonal stressors on the job [12] and it is an extreme and special form of stress response [13]. In recent years, based on the JD-R model, many scholars have discussed specific job demands and job resources that play key roles in occupations, such as civil servants [14], emotional labors [15], and employees of health care institutions [16]. Some scholars have also studied the impact of job characteristics on job burnout, work engagement [17], and emotional exhaustion [18], but there are few studies on the impact of stress response. Police stress response refers to a series of abnormal psychological, physiological and behavioral response of the police due to excessive pressure, such as gastrointestinal discomfort, nervous tension, irritability. Police officers have a wide range of work and high social expectations, as well as different job characteristics from other occupations [19]. Therefore, it is necessary to determine the impact of job characteristics on police stress response.

The JD-R model includes a health-damage process related to job demands. It is assumed that high job demands will consume workers’ resources and encourage work stress [13]. Job demands of police officers mainly refer to the various work scenarios faced by the police that consume energy, such as the work content that brings about work stress, complexity of tasks and other issues. Its operational indicators include quantitative demands, skill demands and emotional demands. As perceptible work stressors, job demands may consume resources in the process of employees’ coping, which may also bring more stress and form a loss helix [16]. Some studies divide the stressors of the police officers into stress from operation and stress from organization[20], The police officers are accompanied by operational risks due to the high risk of their posts, and the stress from operations is related to the special duties of the police[21]. In terms of organization, under China’s police management system, “unified leadership and hierarchical management” has enabled public security organs to undertake many tasks outside their functions, and they are also facing double assessment standards and indicator stress. This has resulted in a serious shortage of police forces on the front line and an overload of police work. Therefore, job demands related to operations and organization may be the cause of police stress [22]. Therefore, the research takes job demands as an important factor affecting police stress response and proposes research hypothesis 1 from the negative results of stress response:

- **Hypothesis 1(H1).** *Job demands have a significant positive impact on police stress response.*

The two processes of the JD-R model (the health-damage process of job demands and the incentive process of job resources) are not independent. Job resources can participate in the health damage process of job demands as buffer factors. Compared with other occupations, police officers’ autonomy is low, and as a job resource, job autonomy may buffer the impact of job demands on stress response. Job autonomy refers to the extent to which the police perceive that they can freely arrange their work methods and procedures. According to the conservation of resources theory, individuals usually respond to various stresses in the working environment with their own resources. When they lose resources or the obtained resources cannot mitigate the working stress, they have a stress response [23]. Under the China's police management system, the individual autonomy of officers is limited, which likely increase stress [24]. Therefore, hypothesis 2 is proposed:

- **Hypothesis 2(H2).** *Job autonomy can buffer the impact of job demands on stress response.*

The incentive process of job resources in the JD-R model shows that, in addition to responding to job demands, job resources also have the potential of motivation [25, 26], which can cultivate employees’ willingness to work hard and have the ability to complete tasks (external motivation) and meet basic psychological needs, such as autonomy, competence and relationship needs (internal motivation)[27]. The concept of basic psychological needs comes from the self-determination theory. Based on the self-determination theory, the satisfaction of basic psychological needs is crucial to the results of human resource management [28], while job demands and job resources will affect the satisfaction of basic psychological needs [29, 30]. When employees perceive more job demands, it will negatively affect the satisfaction of their basic psychological needs [31].

Compared with studying the correlation between basic psychological needs and other variables, it can highlight its value more when it is used as a mediator or moderator among variables [32].Although there have been many studies on the prediction of job demands on burnout and stress, the mediating mechanism of this relationship still lacks explanation [33]. “Stressor-influencing variable-stress results” is a commonly used analysis model [34]. The mediating variables of stressor–stress results found in previous studies include work-family conflict [35], psychological capital [34], emotional dissonance [36], and rumination [37]. According to the self-determination theory, the three basic psychological needs of autonomy, competence, and relationships are not only the necessary conditions for individual psychological growth, internalization, and mental health, but also the necessary conditions for individuals to obtain optimal development [38]. The satisfaction of basic psychological needs has a positive predictive effect on individuals’ happiness [39], mental health [32], and successful relationships [40].For police, the basic psychological needs are the sense of control over the environment, the good relationship with others, the sense of control over their own behavior and the psychological freedom that the police feel. The operational indicators include autonomy needs, relationship needs, and competency needs. Police officers with high satisfaction of their basic psychological needs can better adjust to the negative reactions caused by work stress. If basic psychological needs are not met, individuals will seek other ways of compensation, resulting in more serious psychological and physiological problems, including negative emotions such as depression and anxiety and negative coping styles [41]. The continuous frustration of needs can also lead to the reduction of internal motivation and increase the sense of stress and fatigue [40]. Therefore, the satisfaction of police’s basic psychological needs can predict their stress response.

According to the above discussion, it can be inferred that job demands negatively affect the satisfaction of basic psychological needs, the satisfaction of basic psychological needs negatively affects stress response, and job demands positively affect stress response and meet the test conditions of mediating effect. Therefore, research hypothesis 3 is put forward:

- **Hypothesis 3(H3).** *The influence of job demands on police stress response is partially mediated by basic psychological needs being met.*

**Fig. 1:**
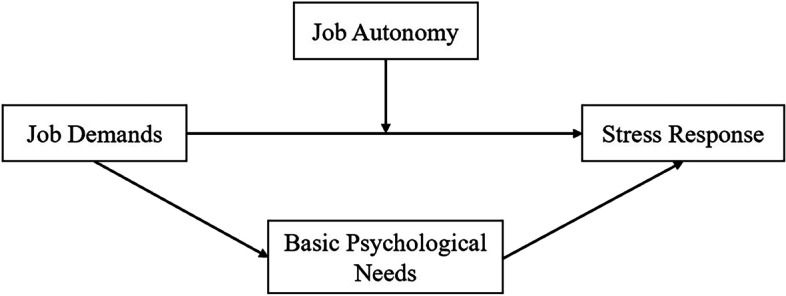
Research Model

We start with the job characteristics of police officers and study the impact of job demands on stress response and the role of job autonomy and basic psychological needs. According to the above theories and research hypotheses, the research model is as follows (Fig. 1):

## Methodology 

### Selection of research sample

The research is aimed at police officers in a public security organization of a municipality, such as security police, criminal investigation police, traffic police, and economic investigation police. A total of 251 valid questionnaires were obtained through a survey. Table 1 highlights demographic information about the respondents that we considered as valid samples. The research data was obtained from questionnaires issued during the training of all front-line police in a district-level public security bureau and there was basically no refusal to investigate. The effective questionnaire reaches 99%. The sample structure conforms to the current job posts ratio and demographic ratio of the police officers.

**Table 1:** The demographic information of the respondents

Particulars	Options	Frequency	Percent
Gender	Male	217	86.45
	Female	34	13.55
	Total (N)	251	100
Age (Years)	26–30	5	1.99
	31–40	119	47.41
	41–50	99	39.44
	51–60	28	11.16
	Total (N)	251	100
Job experience (Years)	< 10	42	16.73
	11–15	66	26.29
	16–20	40	15.94
	21–25	39	15.54
	> 26	64	25.50
	Total (N)	251	100

### Measures

In this study, a Likert 5-point scoring method was used for scale measurement. The respondents need to score each item of each scale from 1 to 5 points in turn, and the scores of each scale are the average scores of all items. All scales were published and widely used. The specific measurements are as follows.

Dependent variable: stress response. At present, there is no occupational stress scale for police officers in China. The stress response part of the occupational stress survey questionnaire for judicial practitioners in Taiwan Guoming Qiu (1994) was used to measure the physiological, psychological, and behavioral stress responses. There are 10 items on the scale. The higher the score, the stronger the respondent’s stress response. The Cronbach, s α coefficient of the scale is 0.938.

Independent variable: job demands. The quantitative demands and skill demands are based on the job demands subscale of the job content scale prepared by Karasek (1998), and the emotional demands are based on the emotional load subscale on the work experience and evaluation questionnaire prepared by van Veldhoven and Meijman (1994). The job demands of police officers were measured using an appropriately modified scale according to the professional characteristics of police officers. There are 13 items on the scale, including five items referring to quantitative demands, four to emotional demands, and four to skill demands. The higher the score, the higher the job demands. The Cronbach’s α coefficient of the scale is 0.900.

Mediating variable: basic psychological needs. The basic psychological needs scale compiled by Deci and Ryan (2001) and translated by Chengfu Yu and other professionals (2012) was used to measure the basic psychological needs of police officers after appropriate correction according to the officers’ professional. The scale includes three subscales of autonomy needs, relationship needs and competency needs with a total of 15 items, including four items referring to autonomy needs, six to relationship needs, and five to competency needs. The higher the score, the better the satisfaction of basic psychological needs. The Cronbach, s α coefficient of the scale is 0.805.

Moderating variable: job autonomy. Job autonomy for police officers was based on the decision autonomy scale compiled by Karasek (1979) and translated by Zhiping Yang (2004). There are 4 items on the job autonomy scale. The higher the score, the higher the job autonomy. The Cronbach, s α coefficient of the scale is 0.898.

### Statistical analysis

The data were statistically analyzed with SPSS22.0, using such tools as the Harman common method deviation test, variable descriptive statistics, correlation analysis and regression analysis among variables. The mediating effect of basic psychological needs and the moderating effect of job autonomy were tested using the plug-in PROCESS of SPSS.

## Results

### Common method deviation test 

The Harman single factor test is used to test possible common method deviations. We found nine factors with eigenvalues greater than 1. At the same time, the variation explained by the first factor is 25.88%, which is less than the critical standard of 40%, indicating that the common method deviation is not obvious.

### Descriptive statistics and related Analysis 

Table 2 shows the min, max, mean, standard deviation and correlation coefficients among the four main variables. The min and max in the table are sample values, and the theoretical max and min of each scale are 5 and 1, that is, in theory, the respondents can get 5 points at most and 1 point at least in each scale. The result shows that the police's job demands score is 3.70. The average of the police stress response was 3.27, and 23.9% of police stress responses scored more than 4 points. According to the survey results of other researchers on the stress response of Chinese police, the result for the grassroots police officers at a municipality's Public Security Bureau is 3.57 [42], the result for the criminal police of a county’ Public Security Bureau is 2.84 [43], and the result for the grassroots young police officers at a municipality's Public Security Bureau is 3.42 [44]. It can be seen that the stress problem of the police group is prominent.

**Table 2:** Descriptive statistics (correlation matrix, min, max, mean, and standard deviation)

	*MIN*	*MAX*	*M*	*SD*	1	2	3	4
1 Job Demands	1.00	5.00	3.70	0.67	-			
2 Job Autonomy	1.00	5.00	2.97	0.91	0.35**	-		
3 Basic Psychological Needs	1.27	4.93	3.30	0.53	0.29**	0.51**	-	
4 Stress Response	1.20	5.00	3.27	0.86	0.13*	-0.20**	-0.44**	-

There was a significant positive correlation between job demands and job autonomy and basic psychological needs (*p* < 0.01). There was also a significant positive correlation between job demands and stress response (*p* < 0.05), and there was a significant negative correlation between job autonomy and basic psychological needs and stress response (*p* < 0.01). Sample size n = 251, *p < 0.05, **p < 0.01, the same below.

### Impact of job demands on police stress responses 

#### Mediating effect test for basic psychological needs

According to the mediating effect test procedure [45] proposed by Zhonglin Wen, we used SPSS22.0 for the multi-level regression test (see Table 3), followed by the macro program PROCESS of SPSS developed by Hayes for the bootstrap test [46]. We repeated the sampling 5,000 times, and analyzed the mediating effect of basic psychological needs between police job demands and stress response (see Table 4). Table 3 shows that job demands and basic psychological needs have a significant impact on stress response, and job demands have a significant impact on basic psychological needs.

**Table 3:** Mediating effect regression test

Variable		Dependent Variable: Stress Response	Dependent Variable: Stress Response	Dependent Variable: Basic Psychological Needs
Control	Gender	-0.22	-0.37*	0.19*
	Age	0.02	-0.09	0.14
	Working Years	-0.03	-0.00	-0.03
Independent	Job Demands	0.36**	0.16*	0.24**
Mediator	Basic Psychological Needs	-0.83**		
*R*^*2*^		0.52	0.21	0.33
*F*		18.58	2.83	7.45

**Table 4:** Bootstrap Results with job demands as independent variables (N = 251)

	Effect	SE	BootLLCI	BootULCI
Total	0.164	0.081	0.004	0.324
Direct	0.361	0.088	0.174	0.524
Indirect	-0.197	0.052	-0.302	-0.096

Through the bootstrap test, we found that the direct effect of job demands on stress response is positive at 0.36, while the indirect effect is negative at -0.20, and the sign symbols were opposite. According to the improved version of the mediating effect test proposed by Zhonglin Wen et al., if the symbols of indirect effect and direct effect are opposite, the total effect will be masked, and its absolute value will be lower than expected [47]. Therefore, the role of basic psychological needs between job demands and stress response should be based on the masking effect, and the effect amount is |ab/c'|= 54.57%. Since the direct effect is significant (the LLCI and ULCI do not contain 0), the basic psychological needs partially mask the main effect. H1 and H3 are true, but the results of the first half of the mediating effect show that job demands positively predict basic psychological needs.

In order to further explore the role of the three sub dimensions of job demands, quantitative demands, skill demands and emotional demands were taken as independent variables, basic psychological needs as mediating variables, and stress responses as dependent variables. The results of the bootstrap test were shown in Table 5–7. When quantitative demands are taken as independent variables (Table 5), the total effect and direct effect are significant, while the indirect effect is not significant, indicating that basic psychological needs do not play a mediating role in the impact of quantitative demands on stress response. When skill demands (Table 6) and emotional demands (Table 7) are taken as independent variables, the direct effect and indirect effect are significant, but the total effect is not significant. And the sign symbols of direct effect and indirect effect were opposite, which is similar to the results when job demands are taken as independent variables (Table 4), indicating that there is an obvious masking effect of basic psychological needs.

**Table 5:** Bootstrap Results quantitative demands as independent variables (N = 251)

	Effect	SE	BootLLCI	BootULCI
Total	0.257	0.055	0.149	0.366
Direct	0.301	0.049	0.205	0.397
Indirect	-0.044	0.031	-0.104	0.020

**Table 6:** Bootstrap Results with skill demands as independent variables (N = 251)

	Effect	SE	BootLLCI	BootULCI
Total	-0.094	0.069	-0.230	0.042
Direct	0.144	0.070	0.006	0.281
Indirect	-0.238	0.056	-0.354	-0.138

**Table 7:** Bootstrap Results with emotional demands as independent variables (N = 251)

	Effect	SE	BootLLCI	BootULCI
Total	0.077	0.072	-0.064	0.218
Direct	0.177	0.065	0.048	0.305
Indirect	-0.099	0.048	-0.198	-0.008

#### Test of the moderating effect of job autonomy 

We checked the moderating effect of job autonomy in the relationship between job demands and stress response. See Table 8 for the results. The interaction item was significant (*p* < 0.05), indicating that job autonomy plays a moderate role in the relationship between job demands and stress response. Among the main effects (Table 8), the coefficient of job autonomy was significant (*p* < 0.01) and negative—that is, job autonomy had a significantly negative impact on stress response.

**Table 8:** Moderating Effect Test

Effect	Dependent Variable: Stress Response	
	Variable	β
Main Effect	Job Demands	0.23**
	Job Autonomy	-0.29**
	*R*^*2*^	0.12
	Adjust *R*^*2*^	0.10
	*F*	6.49
Moderating Effect (adding Interactive Item)	Job Demands	0.29**
	Job Autonomy	-0.32**
	Interactive Item	0.16*
	*R*^*2*^	0.14
	Adjust *R*^*2*^	0.12
	*F*	6.67

**Fig. 2:**
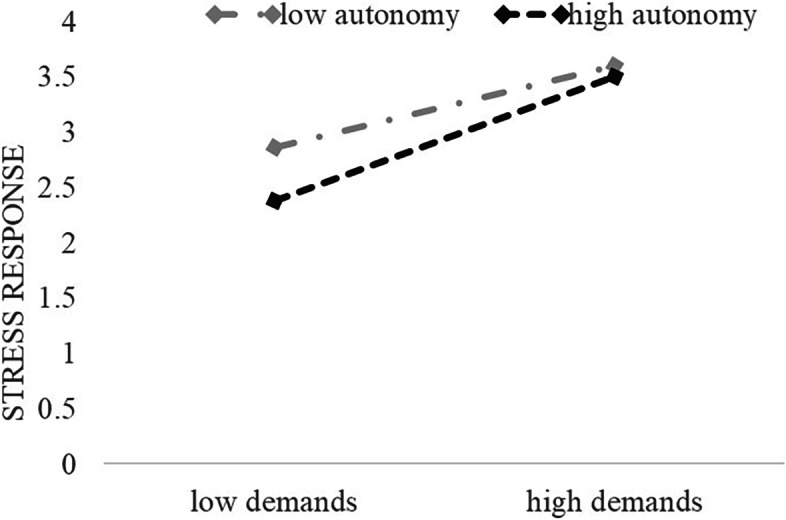
Moderating Effect of Job Autonomy on the Relationship between Job Demands and Stress Response

In order to test the direction of the moderating effect further, a simple slope test was used to analyze the moderation of job autonomy between police job demands and stress response. According to the average score of job autonomy plus or minus one standard deviation, the subjects were divided into a high autonomy level group (sub-jects with one standard deviation higher than the average), a low autonomy level group (subjects with one standard deviation lower than the average), and a medium autonomy level group (subjects between the two groups above). The relationship be-tween job demands and stress response was investigated through grouping regression. The results are shown in Fig. 2 (Fig. 2): with the increase in job autonomy, the positive predictive effect of job demands on stress response is gradually enhanced (from β = 0.19, *p* < 0.05 to β = 0.55, *p* < 0.01). Thus, Hypothesis 2 is not tenable.

## Discussion

The working characteristics of police are different from those of other professions. Chinese police officers are under higher professional stress because of their administrative management system. Taking the job stress of Chinese police officers as the starting point and taking 251 police officers of a Chinese municipality as a sample, in this research we mainly discuss the impact of job demands on the stress response of police, and we verify the moderating effect of job autonomy and the mediating effect of basic psychological needs.

First, there is a double-edged effect for job demands on police stress response. On one hand, job demands increase the stress response of police officers. This is consistent with previous research results. As early as 1988, some researchers found that job demands have a decisive impact on stress responses such as depression, anxiety, and burnout, especially when employees lack autonomy or job control [48]. Job demands require police officers to consume physical and mental resources to cope, resulting in physiological, psychological and behavioral reactions that affect life and work due to resource depletion. On the other hand, job demands can partially reduce police stress response through the masking effect of basic psychological needs. This masking effect is mainly reflected in the impact of police job demands on the satisfaction of basic psychological needs. The results show that the satisfaction of police basic psychological needs can negatively predict stress response, which is consistent with the theory of basic psychological needs. When the working environment meets the basic psychological needs of police officers, it can promote the internalization of the officers' external motivation, thus resulting in a more positive psychological state and work behavior. Different from the results found in previous research, the job demands in this study did not hinder the satisfaction of the basic psychological needs of the police, but improved the degree of satisfaction, resulting in positive results. In fact, many studies have proven that job demands can also play a positive role. Researchers divide job demands into hindrance and challenging demands [49]. Hindrance job demands are the demands that have a negative impact on individuals such as role ambiguity and role conflict, while challenging job demands are the demands that may promote personal growth and realization such as time stress and responsibility [50].

Some researchers have proposed that job demands, as a stressor, whether challenging or a hindrance, need to consume certain resources and energy of employees [51], which will lead to a stress response such as anxiety or fatigue [40]. It may be be-cause challenging stress is a mixed relationship of positive and negative emotional experience. If some challenging stressors bring more negative emotions than positive emotions, or their positive effects are offset by hindrance stressors, individuals will produce more negative stress responses [52]. Some scholars have also found that although some challenging job demands will also have negative effects due to the consumption of energy, they are more positive, such as high job responsibilities and scope or job complexity, which is a potential opportunity to promote individual growth, learn, and gain benefits. Moderate levels of challenge demands may train employees' resilience in future stress events [53]. Employees also have a direct impact on increasing happiness by formulating challenging job demands [54]. Job demands will generally increase the stress response of the police officers, which may be that the positive effects of challenging demands can only partially offset the negative effects of hindering demands. The results of this study may also be related to the professional particularity of police officers. As the public servants of the people, Chinese police officers serve the people wholeheartedly. They have a high sense of professional be-longing, a sense of responsibility [55], noble professional cultivation, and pursuit of their own values, so that their demands for themselves are higher than those of ordinary professional employees.

According to the results, previous research and the actual situation of police work, the satisfaction of basic psychological needs through job demands may mainly be based on skill demands that are challenging, bringing more potential benefits to make up for the loss of individual resources. Studies have proven that skill diversity helps to improve the satisfaction of basic psychological needs [56]. Compared with quantitative demands and emotional demands, skill demands receive more attention in police selection and training. Therefore, when facing skill demands, police officers have strong coping abilities. They can increase their psychological competence, meet their competence needs, strengthen their self-identity, and meet their independent needs. It further promotes the development of social relations and the satisfaction of relationship needs. Moreover, in the sample of Chinese prison guards quantitative demands and emotional demands will increase their job burnout, but skill demands will reduce that burnout [57].

Second, job autonomy plays a moderate role between job demands and police stress response. On one hand, job autonomy negatively affects stress response in police. On the other hand, police job autonomy does not buffer the impact of job demands on stress response but enhances the intensity of this impact. Most studies support the buffering effect of job resources, but some researchers have found similar results. High social support will lead to an increase rather than a decrease in the negative impact of stress [58]. This phenomenon is called the counter-buffering effect, and some re-searchers have found that social support also has a counter-buffering effect on the police officers [21]. Therefore, according to previous research results, job autonomy also has a similar counter-buffering effect in the sampling of police officers—that is, the police officers with high job autonomy will have a stronger stress response caused by their job demands than the police officers with low job autonomy. Moreover, in the study of prison guards, the negative correlation between autonomy and burnout was not supported [57], indicating that the role of autonomy is special for police officers. On one hand, under the highly centralized police management system of China, police work is related to life safety and practical interests of the people. In order to ensure the efficiency and smooth completion of work tasks, police are required to strictly obey the instructions and orders of the Party Central Committee and superiors, and their autonomy is low. This is conducive to the efficiency of task completion. In the case of high autonomy, the job demands are also high, and the individual will bear high work stress, resulting in a negative stress response. On the other hand, as far as the Chinese police group is concerned, strong guidance is required in the case of high demands. The in-dividual will also bear high work stress if high job demands are put forward and high autonomy is given, but the guidance from the superior is not enough and the subordinate lacks the ability to solve problems.

## Conclusions and significances

According to the research results, it can be concluded that JD-R model has special results when applied to the police officers. Job demands will enhance the stress response of the police officers but will be masked by basic psychological needs in the process. As a job resource, job autonomy does not play a moderating role in the impact of job demands on the stress response but increases the impact. Therefore, in order to reduce the stress response of the police officers, we can start with adjusting and optimizing the proportion of police job demands and job autonomy, and we should give high guidance under high demands.

The theoretical significance of the study includes enriching the verification of JD-R model in different occupations, and the research on the mediating effect between job demands and stress response. In practice, it can enhance the attention to police stress and provide theoretical basis for reducing police stress response.

## Recommendations

Healthy psychology and a good mental state are important requirements for police officers to perform their duties and complete their tasks. It is necessary to find measures to reduce the stress response in police through a variety of ways. Otherwise, when work stress becomes chronic, it will strongly affect their physical and mental health [21]. According to the previous research results, in order to reduce the stress response impact on the physical and mental health and work of the Chinese police officers, on the one hand, it is necessary to appropriately adjust the degree of job demands, stimulate the satisfaction of the basic psychological needs of the police officers with reasonable job demands, and prevent the demands and resources caused by excessive job demands from being unequal. In order to reduce the demands of police work, we can adjust the internal management mechanism, optimize the department and task indicator setting to relieve the stress of personnel and task indicators. We also need to implement the police vacation system to ensure that the police officers can be timely rested after the highly loaded work and improve the construction of hardware facilities to liberate the vast number of police officers from the work of "over time, over physical strength, and over intelligence". On the other hand, while properly improving the autonomy of the police officers, the superiors should give sufficient guidance. Neither lack of job resources due to low job autonomy nor stress consequences due to improved job autonomy. Finally, we can start from improving the satisfaction of the basic psychological needs of the police officers and find measures to reduce the stress on the police work, such as creating a harmonious and friendly police team culture to optimize the interpersonal environment and optimizing the police training system to improve the police law enforcement ability and job competency.

## Limitations and future directions

This study has two limitations. First, it is impossible to determine whether external factors will affect the degree of basic psychological needs, and the individual differences of the police officers may also affect the research results. Second, the study did not include mental health variables into the analysis, and stress response cannot directly equate with mental health. Therefore, it can be studied in the future whether the relationship between job demands and stress response can be derived to broader mental health.

## Data Availability

The data presented in this study are available upon request from the corresponding author.
